# Structured reporting of neuroendocrine tumors in PET/CT using [^18^F]SiTATE - impact on interdisciplinary communication

**DOI:** 10.1038/s41598-025-88999-x

**Published:** 2025-02-08

**Authors:** Anna Hinterberger, Lukas Trupka, Sophia Kortbein, Ricarda Ebner, Nicola Fink, Matthias F. Froelich, Dominik Nörenberg, Carmen Wängler, Björn Wängler, Ralf Schirrmacher, Adrien Holzgreve, Matthias Brendel, Stefanie Corradini, Christoph Auernhammer, Johannes Rübenthaler, Freba Grawe

**Affiliations:** 1 DKFZ Hector Cancer Institute at the University Medical Center Mannheim, Im Neuenheimer Feld 280, 69120 Heidelberg, Germany; 2 Department of Clinical Radiology and Nuclear Medicine, Medical Faculty Mannheim, University Medical Center Mannheim, Heidelberg University, Mannheim, Germany; 3 Department of General, Visceral and Transplant Surgery, University Hospital, LMU Munich, Munich, Germany; 4 Department of Radiology, University Hospital of Munich, LMU Munich, Munich, Germany; 5 Biomedical Chemistry, Clinic of Radiology and Nuclear Medicine, Medical Faculty Mannheim, Heidelberg University, Heidelberg, Germany; 6 Research Campus M2OLIE, Medical Faculty Mannheim, Heidelberg University, Heidelberg, Germany; 7 Molecular Imaging and Radiochemistry, Clinic of Radiology and Nuclear Medicine, Medical Faculty Mannheim, Heidelberg University, Heidelberg, Germany; 8 Division of Oncological Imaging, Department of Oncology, University of Alberta, Edmonton, Canada; 9 Department of Nuclear Medicine, LMU University Hospital, LMU Munich, Munich, Germany; 10 DZNE-German Center for Neurodegenerative Diseases, Munich, Germany; 11 Munich Cluster for Systems Neurology (SyNergy), University of Munich, Munich, Germany; 12 German Cancer Consortium (DKTK), Partner Site Munich, a Partnership Between DKFZ and Ludwig-Maximilians-Universität München (LMU), Munich, Germany; 13 Department of Radiation Oncology, University Hospital Munich, LMU Munich, Munich, Germany; 14 Department of Medicine IV, University Hospital of Munich, LMU Munich, Munich, Germany

**Keywords:** Neuroendocrine tumor, PET/CT, Structured reporting, Interdisciplinary communication, Somatostatin receptor, Molecular medicine, Oncology

## Abstract

Our retrospective single-center study aims to evaluate the impact of structured reporting (SR) using a self-developed template on report quality compared to free-text reporting (FTR) in [^18^F]SiTATE Positron Emission Tomography/Computer Tomography (PET/CT) for the primary staging and therapy monitoring of patients diagnosed with neuroendocrine tumors (NET). In total 50 patients were included. FTRs and SRs were generated post-examination. All reports were evaluated by a radiologist and a surgeon through a questionnaire to determine their contribution to facilitating clinical decision-making and to assess their completeness, linguistic quality, and overall quality. SR significantly increased the capacity of facilitating therapy decision-making from 32% in FTR to 55% in SR (*p* < 0.001). Trust in the report was significantly higher in SR with a mean of 5.0 (SD = 0.5) vs. 4.7 (SD = 0.5) for FTR (*p* < 0.001). SR received significantly higher mean ratings regarding linguistic quality with 4.7 for SR vs. 4.4 for FTR (*p* = 0.004) and overall report quality with a mean of 4.9 for SR vs. 4.6 for FTR (*p* < 0.001). Concluding that SR enhances the overall quality of reports in [^18^F]SiTATE-PET/CTs for NET staging, serving as a tool to streamline clinical decision-making and enhance interdisciplinary communication in the future.

## Introduction

Neuroendocrine tumors (NETs) are rare malignancies predominantly originating from the endocrine tissue of the gastroenteropancreatic and bronchopulmonary tract^1,2^. Given the heterogeneous nature of NETs, an accurate assessment of tumor localization, extent, and metastases is crucial for treatment planning (e.g., surgery, peptide receptor radionuclide therapy (PRRT)) to optimize patient´s outcome^3–5^. NETs express a high density of somatostatin receptors (SSTR), which enables their imaging with radiolabeled somatostatin analogs^6–8^. Currently, the most sensitive method for the diagnostic imaging of NETs is somatostatin receptor positron emission tomography/computed tomography (SSTR-PET/CT) and has been endorsed by various guidelines^9–13^. Consequently, ^68^Ga labeled somatostatin analogs such as DOTATOC and DOTATATE have been established as the gold standard for primary staging, restaging, and therapy planning of SSTR-expressing tumors^10,11,14^. The introduction of the novel SSTR-ligand [^18^F]-Silicon-Fluoride-Acceptor (SiFA)-TATE ([^18^F]SiTATE), a ^18^F-labeled somatostatin analog, is gaining increasing relevance in clinical use. This is due to its longer half-life, cost-effective production, and improved tumor-to-background ratios compared to ^68^Ga-radiolabeled tracers^14,15^.

A key element in the diagnosis of NETs is the written report that harmonizes the imaging results of PET and CT, enabling accurate communication between referring/treating clinicians and nuclear medicine physicians/radiologists. To achieve optimal report quality, radiological and nuclear medicine societies recommend using standardized reporting procedures^16–19^, as several comparative studies have demonstrated the superiority of structured reporting (SR) over free-text reports (FTR), not only in terms of completeness and extraction of important clinical information but also in evaluations of linguistic quality and readability^20–24^. In the field of nuclear medicine, first attempts have been made to standardize the acquisition and analysis of molecular imaging, and structured systems have been established to standardized reporting of individual NET lesions using Reporting and Data Systems (RADS) for SSTR-PET/CT^25,26^. In 2018, the European Neuroendocrine Tumor Society (ENETS) developed a standardized report template for the staging of NET patients based on the experience of an interdisciplinary team^25^. However, to date, no study has validated the SR template for the diagnosis of NET by SSTR-PET/CT for clinical use.

Therefore, this study aims to investigate whether the use of SR for SSTR-PET/CT in patients with NET can improve patient management and interdisciplinary communication. We hypothesize that SR will positively influence clinical decision-making and streamline the extraction of information from the examinations. Additionally, we will examine the quality of the reports and assess the physician’s confidence using SR.

## Methods

### Study design

This retrospective study was approved by the local Ethics Committee. Data was collected from an institutional database following the principles of the Declaration of Helsinki and its subsequent amendments. All patients gave written consent to undergo [^18^F]SiTATE PET/CT according to the regulations of the German Pharmaceuticals Act § 13(2b). Fifty patients diagnosed with NET who underwent [^18^F]SiTATE PET/CT for staging and restaging were randomly selected between February 5, 2020, and March 9, 2023.

### Image acquisition

[^18^F]SiTATE-PET/CTs were acquired on Siemens Biograph mCT flow or Siemens Biograph 64 (Siemens Healthineers, Erlangen, Germany). Scans were performed approximately 90 min after intravenous injection of 232 ± 97 MBq [^18^F]SiTATE. PET was acquired with a 2.5 min per bed position. Based on CT scans for attenuation correction, PET images were reconstructed iteratively, with a transaxial 200 × 200 matrix using TrueX (including TOF, 2 iterations, and 21 subsets) with Gaussian post-reconstruction smoothing (2 mm full-width at half-maximum).

Contrast enhanced CT scans, including the neck, thorax, abdomen, and pelvis were performed in *n* = 45 (Imeron 350 mgI/mL, 2.5 mL/s, Bracco Imaging; 1.5 ml/kg body weight). Five patients did not receive contrast agent due to contraindications. All PET/CT scans were analyzed and evaluated using VISAGE PACS Viewer (Visage Imaging GmbH, Berlin, Germany).

### FTR and SR

For FTR (assessed in clinical routine), [^18^F]SiTATE-PET/CT examinations were reported by a resident in nuclear medicine and radiology, respectively, with rather lower levels of experience (up to 2–3 years of experience in hybrid imaging). These reports were then validated by a board-certified radiologist and nuclear medicine physician, respectively (> 7 years of experience in hybrid imaging).

For SR (assessed additionally for the study), a template was developed using a dedicated software (Smart Reporting GmbH, http://www.smart-reporting.com)^27–30^, incorporating control elements such as yes/no options and single/multiple selections. The goal was to create a template specifically applicable for NETs in SSTR-PET/CT staging, which, in theory, could also be used for other tumor entities that can be examined using SSTR-PET/CT. [^18^F]SiTATE-PET/CTs were subsequently re-reported by one reader, blinded to the FTRs using this template.

**Fig. 1:**
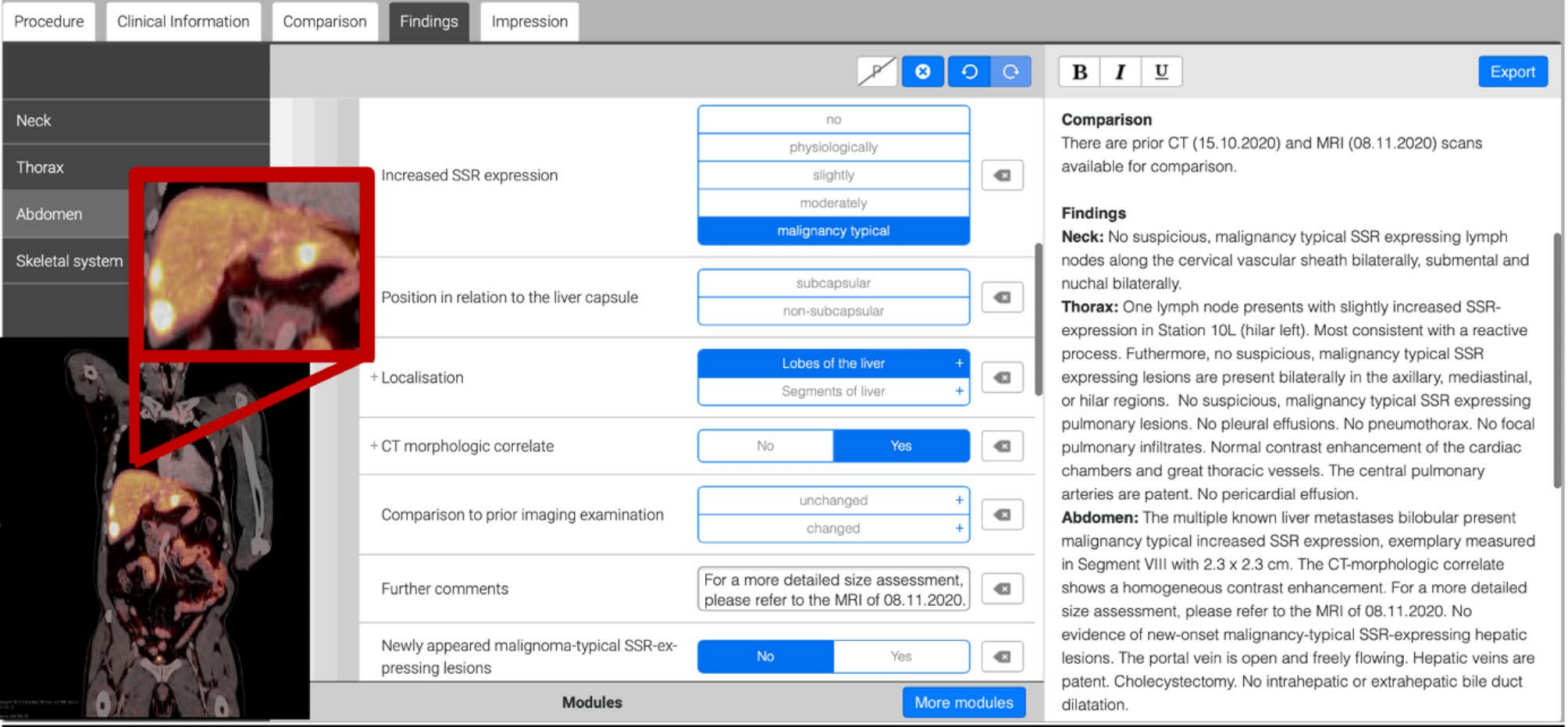
Self-constructed Smart Reporting (Smart Reporting GmbH, http://www.smart-reporting.com) template. Description of liver metastases (a) by stating all findings in the template (b) resulting in an associated coherent text (c).

The SR is divided in 5 sections:^1^ Procedure^2^, Clinical Information^31^, Comparison^32^, Findings, and^33^ Impression. In the “Procedure” section, the applied tracer and dose, the CT contrast agent, and the date of the examination are recorded. The “Clinical Information” section includes patient information and relevant disease history provided by the referring physician at registration. The “Comparison” section lists previous examinations for reference. For the “Findings” section, text modules are generated based on the clinic’s internal free-text report formulations. Sentence elements are combined to create a coherent text with the structure validated by an experienced radiologist (J.R). These elements are based on recommendations of ENETs and the latest guidelines in oncological imaging, such as the international association for the study of lung cancer (IASLC)^24,34^. The findings are described in detail, including whether the pathology is known from previous examinations or newly detected, the number and localization of metastases, description of recurrent tumor, and SSTR expression (none, physiological, low, moderate, and typical for malignancy; Fig. 1*)*. If additional information has to be added, there is the option to include “further comments” in form of a free-text.

**Fig. 2:**
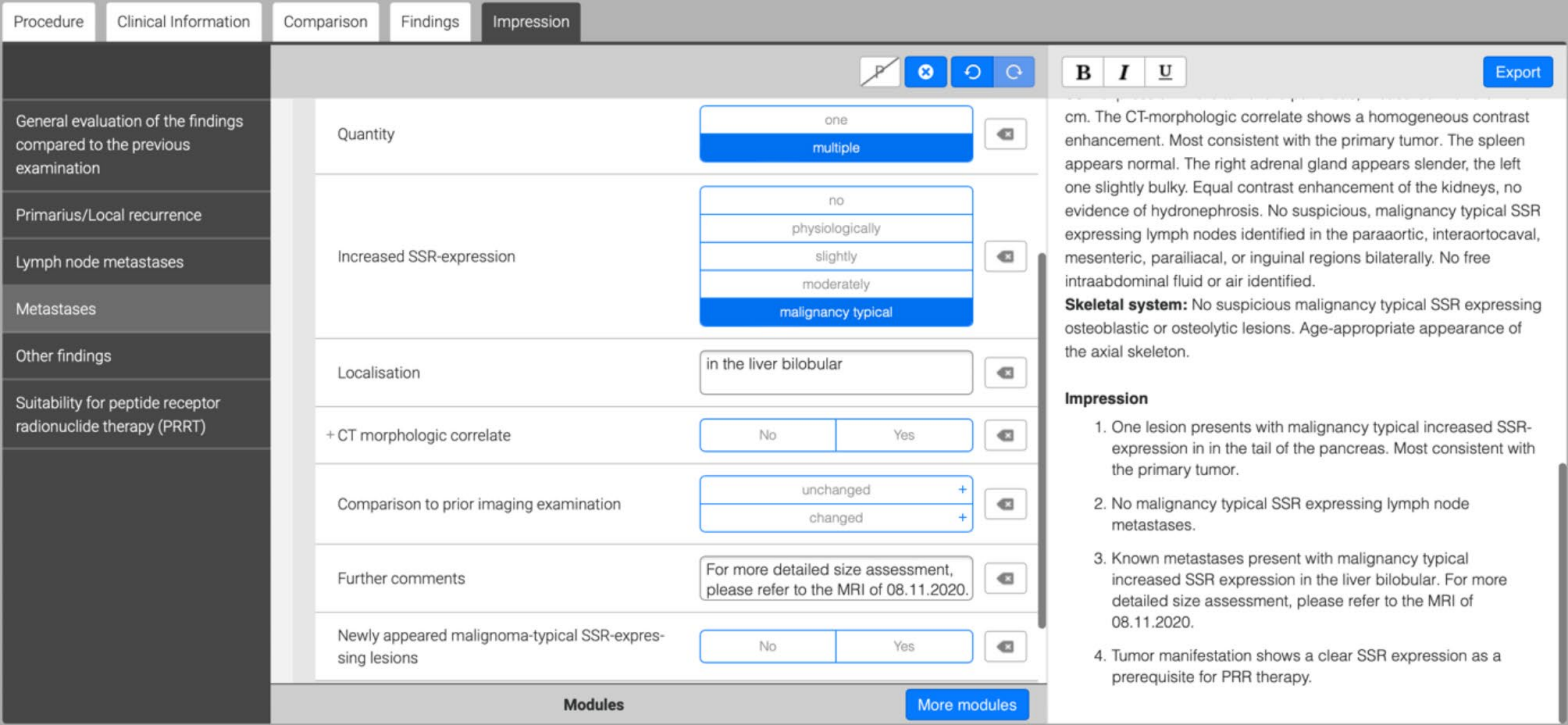
Self-constructed Smart reporting (Smart Reporting GmbH, http://www.smart-reporting.com) template. Description of the overall impression of metastases (a) by stating the impression in the template (b) resulting in an associated coherent text (c).

In the “Impression” section, an overall assessment according to the “tumor, node, metastasis” (TNM) classification is provided^35^. Additionally, an assessment of whether the tumor manifestation is suitable for PRRT is included (Fig. 2).

### Report evaluation

50 patients diagnosed with NET whom underwent [^18^F]SiTATE-PET/CT between February 5, 2020, and March 9, 2023 got randomly selected and included. A questionnaire was designed to evaluate both FTR and SR. It aimed to determine whether all key questions were answered and to identify any missing information. Additionally, the questionnaire assessed the report´s impact on clinical decision-making and information extraction, and evaluated the completeness of the report. Using a 6-point Likert scale (1 = insufficient, 6 = excellent), the quality of language, overall report quality, and clinicians’ confidence in the report were rated.

**Fig. 3:**
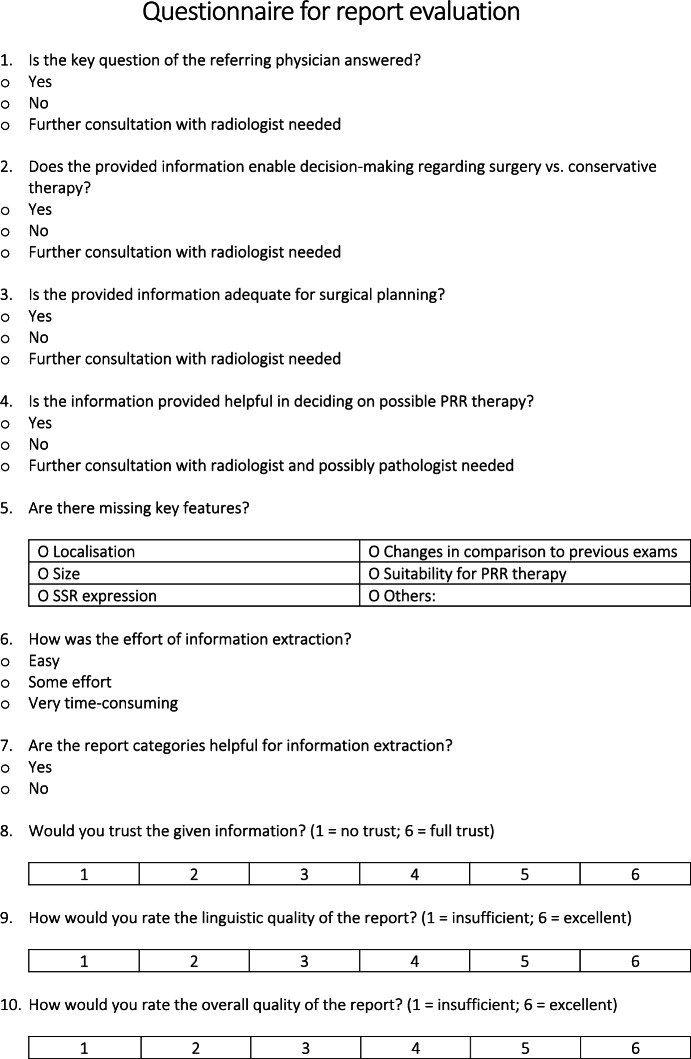
Self-developed questionnaire.

A total of 100 reports (50 FTRs and 50 SRs), along with a questionnaire for each report were sent to two physicians: one with 3 years of experience in radiology and nuclear medicine (A.H) and the other with 3 years of experience in visceral surgery (L.T). The questionnaires were completed independently and in a blinded manner. The questionnaire is presented in Fig. 3.

### Statistical analysis

Statistical analyses were conducted using IBM SPSS Statistics Version 25 (Armonk, New York, NY, USA). Binominal data were compared using the McNemar test, while the significance of responses on the Likert scale was assessed with the Wilcoxon-Signed-Rank test. Interrater reliability was measured with the Cohen’s Kappa test. The significance level was set at *p* = 0.05.

### Sample size calculation

For our calculations, we examined the results of other studies with similar study design and question. A completeness rating of high or very high (> 80%) was assumed in 55% of the FTR and 70% of the SR^20–22^. To statistically verify this assumption with a power of 80% and a significance level of *p* = 0.05, a sample size of *n* = 82 (41 FTR, 41 SR) was required. To ensure that the effect size is not overestimated, the number of cases in the study was increased to *n* = 100.

## Results

In our study, we included 50 patients diagnosed with NET, all of whom underwent [^18^F]SiTATE-PET/CT between February 5, 2020, and March 9, 2023. The summary of patients charcatersitsics is shown in Table 1.

**Table 1:** Patients’ characteristics of 50 patients diagnosed with NET.

Patients characteristics	Total counts (*n*)
men	27
women	23
Mean age (years)	63
Initial staging	5
Re-staging^1^	45
surgery	14
biotherapy	10
chemotherapy	3
combinational therapy^2^	18
PD^3^	9
RD^4^	8
SD^5^	27
Mixed response	1
Primary gastroenteropancreatic	20
Ileum	15
Small intestine	2
Coecum	1
Stomach	2
Lung	6
Other	30
No metastases	15
Metastases	35
Lymph node metastases	4
Distant metastases	13
Both	18

^1^Re-staging is defined as a PET/CT scan performed during ongoing therapy or was performed after therapy/surgery.

^2^Including a combination of PRRT, surgery and/or biotherapy, and/or chemotherapy and/or radiation therapy.

^3^Progressive disease.

^4^Regressive disease.

^5^Stable disease.

Both reviewers completed 50 questionnaires on SR and 50 questionnaires on FTR, totaling 100 SR and 100 FTR cases. All 200 questionnaires were filled out completely by the 2 reviewers.

Key questions of the referring physicians were answered in 94% of SR vs. 87% of FTR (*p* = 0.143). The information provided was significantly sufficient for decision-making (based on guideline recommendations^1,5^) regarding surgery vs. conservative therapy in 55% of SR vs. 32% of FTR (*p* = 0.001) and information was adequate for surgical planning in 50% of SR vs. 48% of FTR (*p* = 0.824). Furthermore, SR was significantly helpful in the decision for PRRT therapy in 95% of reports vs. 80% in FTR (*p* = 0.003). In question 1–4 the option “further consultation needed with radiologist” or “further consultation needed with radiologist or pathologist” was not chosen in FTRs or SRs.

Using SR significantly increased report completeness by lowering the key feature miss-rate, at least one key feature was missing in 51% of FTR vs. 11% of SR (*p* = 0.001). The most frequently missed key feature in the SRs was the suitability for PRRT (*n* = 7). Information extraction was considered easy in 48% of SR vs. 45% of FTR (*p* = 0.668). In 7% of SR cases, information extraction was considered time-consuming compared to FTR (*p* = 0.668). The report structure was helpful in 100% of both SR and FTR.

**Fig. 4:**
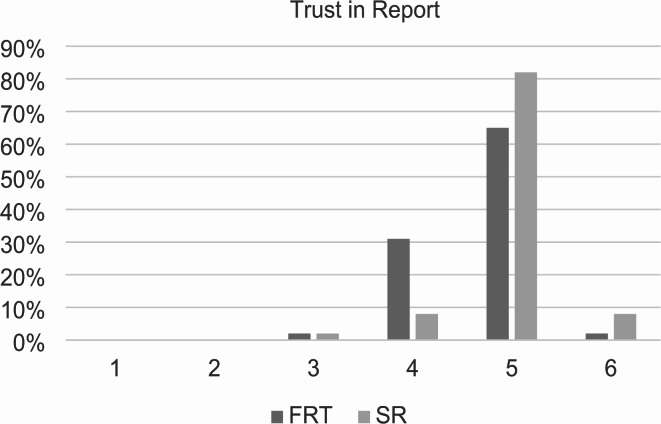
Trust of referring physicians in the report. Structured reports and free-text reports were rated based on a Likert scale ranging from 1 to 6 (1 = insufficient, 6 = excellent). The diagram illustrates the degree of the trust on the x-axis and the percentage distribution on the y-axis. SR = structured reports, FTR = free-text reports. *p* < 0.001 using Wilcoxon-Signed-Rank test.

**Fig. 5:**
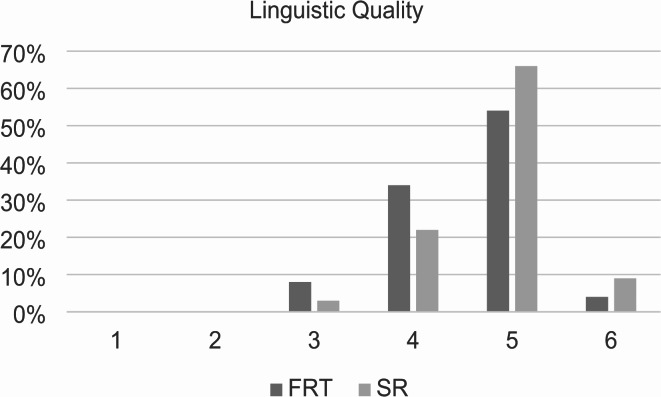
Linguistic quality of the reports. Structured reports and free-text reports were rated based on a Likert scale ranging from 1 to 6 (1 = insufficient, 6 = excellent). The diagram illustrates the degree of linguistic quality on the x-axis and the percentage distribution on the y-axis. SR = structured reports, FTR = free-text reports. *p* = 0.004 using Wilcoxon-Signed-Rank test.

**Fig. 6:**
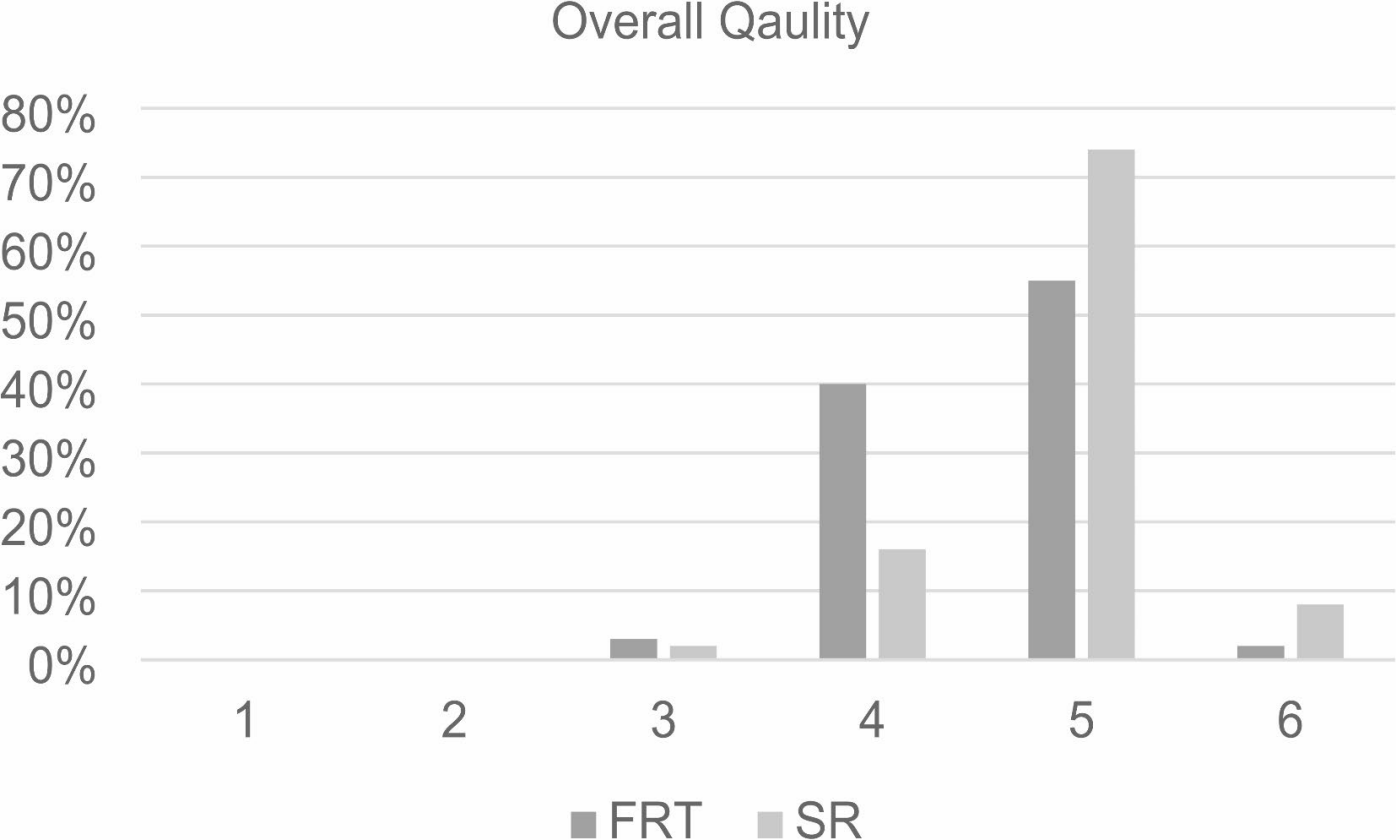
Overall quality of the reports. Structured reports and free-text reports were rated based on a Likert scale ranging from 1 to 6 (1 = insufficient, 6 = excellent). The diagram illustrates the overall quality of the reports on the x-axis and the percentage distribution on the y-axis. SR = structured reports, FTR = free-text reports. *p* < 0.001 using Wilcoxon-Signed-Rank test.

Overall, SRs received significantly higher ratings on the Likert scale compared to FTR as presented in Figs. 4, 5 and 6. The trust of referring physicians in the report was significantly increased by SR with a mean of 4.96 ± 0.49 (95% confidence interval (CI): 4.86–5.06) vs. 4.67 ± 0.55 (CI: 4.56–5.78) for FTR (*p* < 0.001), shown in Fig. 4. The most frequent chosen number in the scale was 5 with 65% for FTR and 82% for SR.

Linguistic quality of the reports was rated significantly higher in SR with a mean of 4.81 ± 0.63 (CI: 4.96–4.93) vs. 4.54 ± 0.70 (CI: 4.4–4.68) for FTR (*p* = 0.004) (Fig. 5). The most frequently chosen number on the scale for FTR and SR was 5 with 54% for FTR and 66% for SR.

Overall report quality was rated 4.56 ± 0.59 (CI: 4.44–4.68) for FTR vs. 4.88 ± 0.56 (CI: 4.77–4.99) for SR, with a statistically significant difference (*p* < 0.001; Fig. 6). The most common rating for both FTR and SR was 5, with 55% of FTRs and 74% of SRs receiving this score.

## Discussion

Our study is the first to investigate whether a structured narrative report (SR) for staging neuroendocrine tumor (NET) patients with somatostatin receptor PET/CT (SSTR-PET/CT) enhances clinical communication in an interdisciplinary setting. Our results demonstrate that employing SR in SSTR-PET/CT examinations of NET significantly enhances report completeness, treatment planning, linguistic quality, trust in reports by referring physicians, and overall report quality compared to conventional free-text reports (FTR).

Among 50 patients, 45 underwent re-staging to determine disease status (progression, regression, or stability). Radiologists and nuclear medicine physicians typically compare current oncological status with previous images in FTRs. In our study, there was no significant difference in answering the key question from referring physicians (94% for SR vs. 87% for FTR). However, SRs provided more sufficient information for decision-making regarding surgery versus conservative therapy (55% for SR vs. 32% for FTR). These results align with Schoeppe et al., who found SRs significantly more beneficial for clinical decision-making in B-cell lymphoma patients^22^.

Interestingly, our results showed no significant difference in surgical planning adequacy (50% for SR vs. 48% for FTR), despite studies showing SR’s superiority in surgery planning due to clearer, more complete reports^24,36–38^. This discrepancy may arise because most NET patients in our cohort had already undergone surgery or were receiving biotherapy (*n* = 31), thus surgical intervention was not considered for most. SR was significantly helpful for deciding on PRRT therapy (95% of reports vs. 80% for FTR). Molecular imaging RADS, such as SSTR-RADS, focus on assessing individual lesions and further recommendations, leading to better decision-making for PRRT with SRs compared to FTRs^26,39,40^. Even though our goal was to provide an overall oncological impression rather than describe individual lesions, a structured approach simplifies PRRT decision-making.

The goal of providing an overall oncological impression in a narrative report, ensuring no lack of depth of information needed for data extraction or clinical management, aligns with ENETS’ pursuits^25^. While the ENETS report is not yet validated, our self-constructed report, based on ENETS’ proposal, showed that SR prevents from latter mentioned, as key feature miss rates were lower (51% for FTR vs. 11% for SR). This agrees with Brook et al., showing SR for pancreatic cancer CT-staging improved key feature reporting frequency (e.g., tumor location, size, enhancement, node status, and vascular involvement)^24^. Similarly, our study showed higher overall SR quality on a 6-point Likert scale (4.88 for SR vs. 4.56 for FTR), consistent with studies demonstrating structured approaches like SSTR-RADS are highly reproducible and accurate^26,40^. Despite our detailed report, there was no significant difference in the simplicity of information extraction (48% for SR vs. 45% for FTR). However, information extraction from SR was deemed “very time-consuming” in 7% of cases. The time required to create SRs was not quantitatively measured; subjectively, no difference in time was noted between SR and FTR. Previous studies have shown inconsistent results regarding SR time efficiency, and radiologists may need to familiarize themselves with SR tools to optimize their use^20,37,41,42^. Moreover, different templates to create a narrative SR should be compared in follow-up studies. These studies should also take an interdisciplinary approach (including input from nuclear medicine specialists and radiologists) to evaluate time efficiency in report creation and information acquisition. This would facilitate the development of a template that can be applied universally and reciprocally.

The most crucial criterion for effective communication between nuclear medicine specialists, radiologists, and treating physicians is trust in the report. Our study demonstrated increased trust with SR compared to FTR (4.96 for SR vs. 4.67 for FTR on a 6-point scale). Previous studies have shown that SRs improve surgeons’ satisfaction and reduce the perceived need to contact the interpreting radiologist for explanations, indicating more trust in SRs^43,44^. Similar results were shown for prostate MRI, where SRs were rated superior regarding summary quality and overall report satisfaction^45^. Furthermore, it has been shown that unexperienced reporters themselves stated decreased anxiety to report on SSTR-PET/CT when applying structured reporting systems as SSTR-RADS^46^. In previous studies, clinicians rated clarity of language as the second most important component of a radiology report, after diagnostic accuracy^47^. Nonstandard terminology results in considerable variability, leading to potential ambiguity and inaccurate understanding by referring providers as well as decreased adherence to the international guidelines^48,49^. In our study, SRs showed significantly higher ratings in linguistic quality (4.81 for SR vs. 4.54 for FTR).

There is likely to be an increasing trend towards structured reports in nuclear medicine to provide more precise findings and increase clinical acceptance of novel radiotracers in routine practice. However, careful consideration is required in SR development regarding the level of detail and whether prioritizing comprehensive descriptions outweighs making the process more time-efficient. In our template, for example there was even the option to add “further comments” for each finding which makes the report very detailed. The positive outcome for SR in our study cannot be generalized to all SR types used by radiologists, as different formats exist, and the choice of suitable SRs may depend on referring physicians’ preferences.

Although our study was the first to evaluate a complete narrative SR in an interdisciplinary setting for its impact on report quality, further studies are needed to achieve general satisfaction among referring physicians with SRs. Additionally, automated segmentation and calculation of standardized uptake values (SUV) in PET/CTs are advancing and might be integrated into SR tools to further streamline and enhance reporting.

Several limitations must be acknowledged. SRs were not created in clinical practice and not simultaneously with FTR due to the retrospective design. All SRs were created by one reader; future studies should involve various reporting individuals to assess SR stability over FTR. Future evaluations should include multiple readers with different training levels to ensure repeatability and avoid information bias. Most patients included for constructing retrospective SRs underwent re-staging after or during therapy, potentially simplifying reporting compared to initial diagnoses with extensive malignancy spread. Further studies are necessary to evaluate SR in clinical routine with multiple radiologists and nuclear medicine physicians creating SRs, as well as multiple clinicians for report evaluation, to provide additional evidence on the benefits of SR for SSTR-PET/CT examinations.

## Conclusion

In our study we found that a structured assessment of SSTR-PET/CTSs improved trust in reports, clinical decision-making and overall quality due to complete and comprehensive reporting.

## Data Availability

The data that support the findings of this study are not openly available due to reasons of sensitivity and are available from the corresponding author upon reasonable request.

## Abbreviations

CI: Confidence interval
ENETS: European Neuroendocrine Tumor Society
FTR: Free-text reporting
IASLC: International Association for the Study of Lung Cancer
NET: Neuroendocrine tumor
PET/CT: Positron Emission Tomography/Computer Tomography
PRRT: Peptide receptor radionuclide therapy
RADS: Reporting and Data Systems
SD: Standard deviation
SiFA: Silicon-Fluoride-Acceptor
SR: Structured reporting
SSTR: Somatostatin receptors
TNM: Tumor, node, metastasis
